# Predictors and trajectories of ED visits among patients receiving palliative home care services: findings from a time series analysis (2013-2017)

**DOI:** 10.1186/s12904-020-00626-w

**Published:** 2020-08-16

**Authors:** Alberto Borraccino, Sara Campagna, Gianfranco Politano, Marco Dalmasso, Valerio Dimonte, Maria Michela Gianino

**Affiliations:** 1 Department of Public Health and Pediatrics, University of Torino, Via Santena 5 bis, 10126 Torino, Italy; 2 Department of Control and Computer Engineering, Politecnico of Torino, Corso Duca degli Abruzzi 24, 10129 Torino, Italy; 3 Regional Public Health Observatory (SEPI), Local Health Unit TO3, Via Sabaudia 164, 10095 Grugliasco (To), Italy

**Keywords:** Emergency departments, Palliative care, Home care service

## Abstract

**Background:**

Current policies recommend integrating home care and palliative care to enable patients to remain at home and avoid unnecessary hospital admission and emergency department (ED) visits. The Italian health care system had implemented integrated palliative home care (IHPC) services to guarantee a comprehensive, coordinated approach across different actors and to reduce potentially avoidable ED visits. This study aimed to analyze the trajectories of ED visit rates among patients receiving IHPC in the Italian healthcare system, as well as the association between socio-demographic, health supply, and clinical factors.

**Methods:**

A pooled, cross-sectional, time series analysis was performed in a large Italian region in the period 2013–2017. Data were taken from two databases of the official Italian National Information System: Home Care Services and ED use. A clinical record is opened at the time a patient is enrolled in IHPC and closed after the last service is provided. Every such clinical record was considered as an IHPC event, and only ED visits that occurred during IHPC events were considered.

**Results:**

The 20,611 patients enrolled in IHPC during the study period contributed 23,085 IHPC events; ≥1 ED visit occurred during 6046 of these events. Neoplasms accounted for 89% of IHPC events and for 91% of ED visits. Although there were different variations in ED visit rates during the study period, a slight decline was observed for all diseases, and this decline accelerated over time (b = − 0.18, *p* = 0.796, 95% confidence interval [CI] = − 1.59;1.22, b-squared = − 1.25, *p* < 0.001, 95% CI = -1.63;-0.86). There were no significant predictors among the socio-demographic factors (sex, age, presence of a non-family caregiver, cohabitant family members, distance from ED), health supply factors (proponent of IHPC) and clinical factors (prevalent disorder at IHPC entry, clinical symptoms).

**Conclusion:**

Our results show that use of ED continues after enrollment in IHPC, but the trend of this use declines over time. As no significant predictive factors were identified, no specific interventions can be recommended on which the avoidable ED visits depend.

## Background

Today, home care represents the best response to epidemiological changes in the population (aging, increased comorbidity, and chronic pathologies) and the economic sustainability of national healthcare services [1]. This is also true for the delivery of palliative care, the goal of which is to prevent and relieve suffering and provide the best possible quality of life to patients and their families, regardless of their stage of illness [2, 3]. In Italy, palliative care is guaranteed by law (National Law 38/2010) to people with chronic and progressive diseases for which a cure is not available or when complete reversal of the disease and its process is no longer possible; it is not reserved only for persons who are nearing the end of their life. The purpose of palliative care is to assure the patient and those involved in his/her life have an optimal quality of life [4]. Palliative care may be required for a wide range of diseases, such as cardiovascular diseases, cancer, chronic respiratory diseases, multiple sclerosis, dementia, and tuberculosis. Palliative care in Italy is provided in multiple settings, including hospices, hospitals, residential facilities, and at home, referred as integrated palliative home care (IHPC). IHPC services are delivered and administered by Palliative Care Units, which create multi-professional teams that ensure medical, nursing, rehabilitation, and psychological services, as well as social, protective, and spiritual support. After referral by a general practitioner, multi-professional teams decide whether a patient can receive IHPC based on specific, multi-professional assessment scales. IHPC requires the creation of an individual care plan, the purpose of which is to identify the goals of care and the most appropriate interventions in case of problems. This plan is prepared by the multi-professional team and must be shared with the patient and their family and/or caregiver, as it constitutes a therapeutic care contract. The aim of the individual care plan is to guarantee a comprehensive, coordinated approach across different actors, and avoid unnecessary care, hospital admission, and emergency department (ED) visits.

Indeed, although the benefits associated with hospitalization for patients with palliative care needs cannot be denied [5], ED visits are considered an indicator of poor quality in home care services [6]. Moreover, several studies have shown that ED visits in IHPC patients are not essential and potentially avoidable [7–10]. The Italian healthcare system has initiated a shift from institutional palliative care to IHPC, and many efforts have been made to improve the access to and quality of IHPC. In this perspective, it could be useful to evaluate whether patients enrolled in IHPC have reduced ED visits over time and to identify the factors associated with ED use. To the best of our knowledge, there is currently a paucity of epidemiological studies in this area. This study aimed to analyze the trajectories of ED visit rates among patients receiving IHPC in the Italian healthcare system, as well as the association between socio-demographic, health supply, and clinical factors.

## Methods

We conducted a pooled, cross-sectional, time series analysis of ED visit rates among patients receiving IHPC in the period 2013–2017 in the Piedmont Region, which is the second largest region in Italy, with a population of more than 4 million inhabitants over an area of 25,387 km^2^ [11]. A clinical record is opened at the time a patient is enrolled in IHPC and closed after the last service is provided. Every such clinical record was considered as an IHPC event, and IHPC event was used as the unit of analysis. As such, patients may have had one or more IHPC events during the study period; the ED visits analyzed were those that occurred during IPHC events.

The following factors at IHPC entry were considered: the socio-demographic factors sex, age (≤18, 19–65, 66–80, 81–90, 91–100, > 100 years), presence of a non-family caregiver, number of cohabitant family members (0, 1, 2, ≥4), and distance to the nearest ED (≤5, 6–20, > 20 min); the health supply factor proponents of IHPC (general practitioner, hospital, residential facility, other setting); and the clinical factors prevalent pathology at IHPC entry (categorized according to the International Classification of Diseases 9th revision: bone, skin, and breast neoplasms; cardiocirculatory diseases; digestive system diseases; digestive system neoplasms, endocrine and metabolic diseases; lymphatic neoplasms; mental disorders; neurologic disorders; other diseases; other neoplasms; respiratory diseases; respiratory system neoplasms; urogenital diseases) and the reason for ED visit (symptoms of the nervous system, abdominal pain, chest pain, dyspnea, shock, non-traumatic bleeding, trauma, temperature, urological symptoms, rhythm alteration, other symptoms).

All data were taken from two databases that are part of the official Italian National Information System: Home Care Services and ED use. Data from these databases were merged using the universal patient ID number, an anonymous, unique code assigned to each patient that is used for all compulsory registry data within the ministerial system. Data on distance to the nearest ED was obtained from the National Agency for Territorial Cohesion (“Agenzia per la Coesione Territoriale”, part of the “Dipartimento per le politiche di sviluppo e di coesione”), which classifies areas of residence based on the average distance to the nearest hospital with an ED [12].

### Statistical analyses

First, a time trend analysis was performed by computing a linear regression estimate of the annual ED visit rate per patient receiving IHPC. The trend was analyzed for the relationship between ED visit rates and prevalent disorder at IHPC entry. Each linear regression was modelled as a linear and quadratic regression, and each model was tested against a t-Wald test of estimated coefficients and corrected for Robust Standard Errors for Panel Models, using the Beck and Katz Robust Covariance Matrix Estimators. For significative regression, the trend direction was reported as the sign of the beta coefficient. Second, a pooled, cross-sectional, time series analysis with fixed-effect estimation was performed to assess the association between ED visit rates and selected independent factors (socio-demographic, health supply, and clinical factors) over the 5-year study period [13].

One advantage of fixed-effects models is that they control for time-invariant heterogeneity among pathologies by removing the effect of those time-invariant characteristics so the net effect of the predictors on the outcome factor can be assessed. We used the Hausman test to examine each fixed-effect model against a random-effect model [14]. In both the fixed- and random-effect models, the significance of each predictor was assessed by using robust estimators for the standard errors. Standard estimation of predictors was fact corrected with a heteroskedasticity-consistent estimation of the covariance matrix of the coefficient, computed according to the Arellano method [15, 16]. The presence of exogenous time trends in both the dependent and independent factors (i.e., time-fixed effects) was controlled by adding dummy variables to the model for each year of the study period except the first.

To avoid model over-fitting, age was divided into six categories. The relationship between all the remaining dependent and independent factors were examined separately, resulting in seven distinct fixed-effects models. This choice was driven primarily by concerns about model over-fitting and multi-collinearity. The R framework [17] was used to perform all analyses and the significance level was set at *p* < 0.05 for all analyses.

### Ethics statement

The Italian National Information System databases for Home Care Services and ED use are official, anonymized Ministerial Health information systems. All such systems are centrally anonymized and are available to be used for administrative and/or epidemiological studies without any further authorizations. Therefore, ethics committee approval was not required.

## Results

A total of 20,611 patients were enrolled in IHPC during the study period (2013–2017), and they contributed 23,085 IHPC events. There were 18,907 patients (92%) with only one IHPC event, 1264 (6%) with two, and 440 (2%) with ≥3 events. Eigthy-3 % of all IHPC events had a duration of < 100 days.

### Emergency department visit rates

**Fig. 1:**
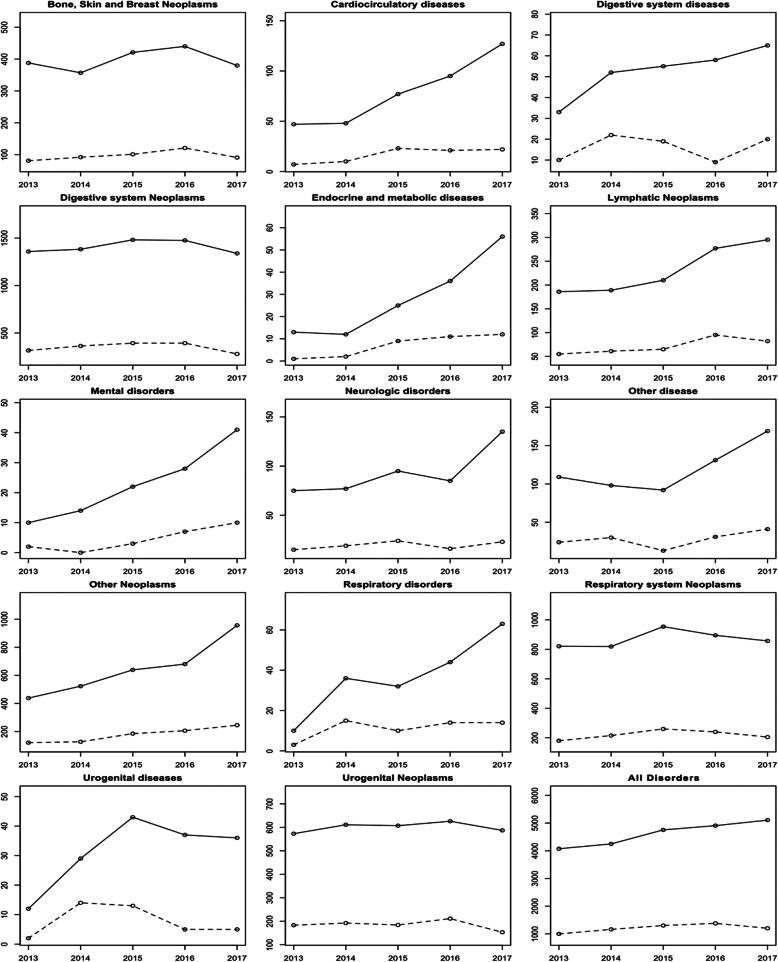
Number of IHPC events and ED visits, by Pathologies at IHPC entry and overall 2013/2017. Solid line: Number of IHPC events over time; Dashed lines - ED visits pattern over time

Of the 23,085 IHPC events during the study period, at least one ED visit occurred in 6046 of them (26 ED visits per 100 IHPC events). Stratification by pathology at IHPC entry shows that most IHPC events were carried out for patients with neoplasms. Indeed, IHPC events for neoplasms accounted for approximately 89% of all IHPC events during the 5-year period. Consequently, neoplasms were responsible for 91% of all ED visits (Fig. 1).

There was great variability in ED visit rates across pathologies, and no rate fell below 10% throughout the 5-year study period. Patients with neoplasms showed less variability in ED visit rates, with values ranging from 20 to 35%. Two pathologies, digestive system diseases and respiratory diseases, had ED visit rates of over 30% in almost all the years analyzed (Fig. 1).

### Annual trends in emergency department visit rates

**Fig. 2:**
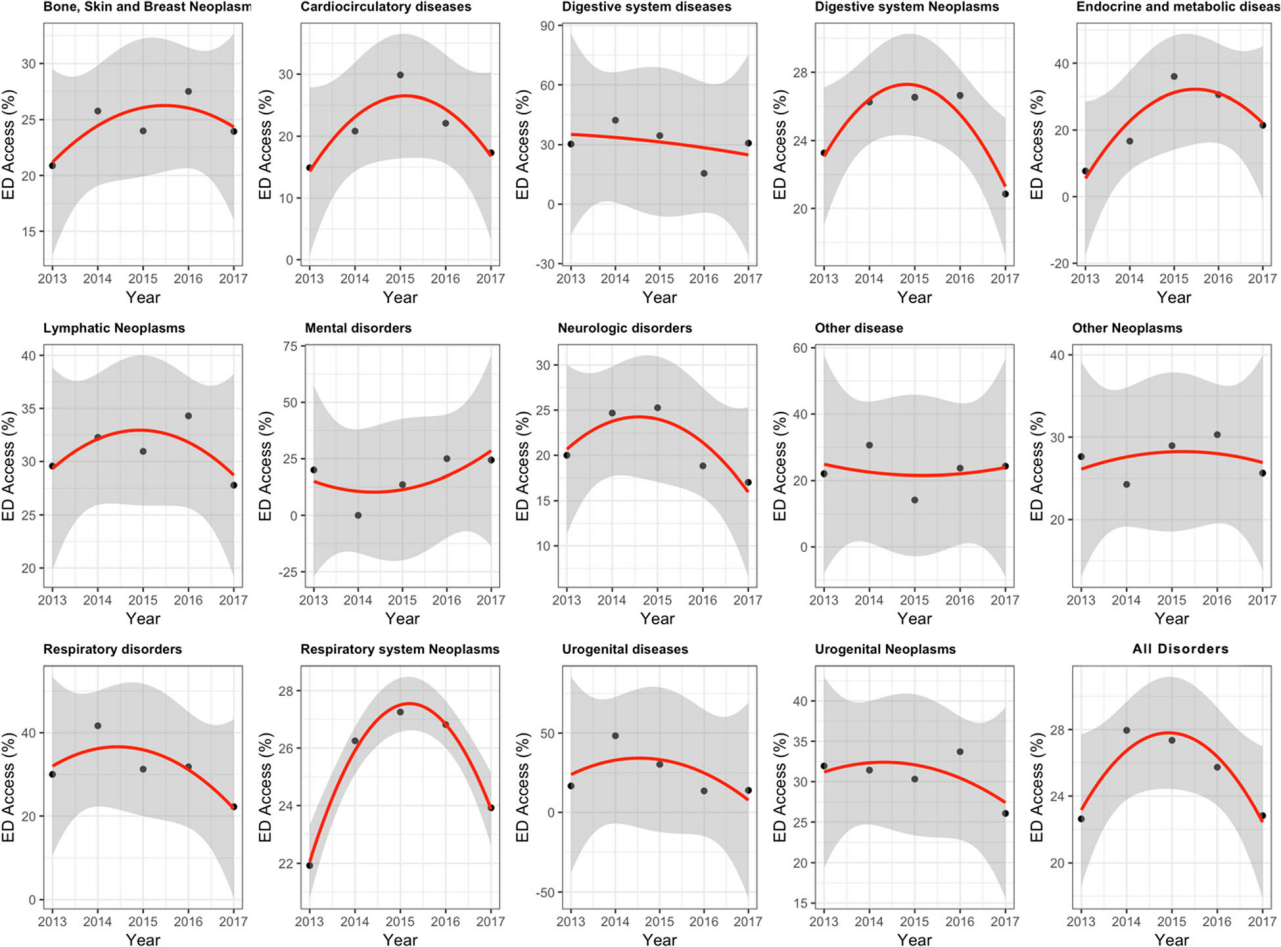
The fixed-effects regression analysis. ED visit rates (%), by pathologies at IHPC entry and overall, 2013/2017

The linear trend of ED visit rates was not significant (coefficient for the linear term = − 0.18; 95% confidence interval [CI] = − 1.59;1.23) but was significantly “curved” (coefficient for the quadratic term: -1.25; 95% CI = -1.64;-0.86). When the coefficient for the linear term is negative and near zero, and the coefficient for a quadratic term is significant and more negative, from a mathematical perspective, it results in a convex time trend, so that the value at the end of the study period is lower than that at the beginning. This means that the average annual ED visit rate decreased over the 5-year study period, and that this decrease accelerated over time (Fig. 2).

Unexpected visit rates by pathology were observed in the fixed-effects model (Fig. 2). Some pathologies had a convex trend with a slight decrease, such as urogenital diseases, digestive system diseases, respiratory diseases, neurologic disorders, urogenital neoplasms, and digestive system neoplasms, whereas lymphatic neoplasms, cardiocirculatory diseases, other neoplasms, respiratory system neoplasms, bone, skin, and breast neoplasms, and endocrine and metabolic diseases had a convex trend with a slight increase during the 5-year study period. The convex trend demonstrates that all pathologies experienced a reverse trend in ED visit rates or an acceleration in the decrease of ED visit rates. Only two pathologies, other diseases and mental disorders, had a concave trend.

ED visit rates declined more slowly among patients with neoplasms (coefficient for the linear term = − 0.0157, 95% CI = -2.3514;2.3199 and coefficient for the quadratic term = − 0.9591, 95% CI = - 2.4318;0.5135) than in those with other pathologies.

### Factors and emergency department visit rates

Regression analyses on the impact of socio-demographic, health supply, and clinical factors on ED visit rates showed no significant results (Table 1).

**Table 1:** Results of the regression analysis

***Regressor***	***Rate of ED visits***
Distance to the nearest ED: Less than 5 min	−1.194
	(0.960)
Distance to the nearest ED*:* From 6 to 20 min	−1.431
	(0.771)
*Observations*^*#*^	*70*
*R*^*2*^	*0.128*
*Adjusted R*^*2*^	*−0.114*
*F Statistic*	3.978^**^ *(df = 2; 54)*
Male	0.194
	(0.145)
*Observations*^*#*^	70
*R*^*2*^	0.033
*Adjusted R*^*2*^	−0.213
*F Statistic*	1.900 (df = 1; 55)
Presence of a non-family caregiver: YES	0.018
	(0.071)
*Observations*^*#*^	70
*R*^*2*^	0.001
*Adjusted R*^*2*^	−0.253
*F Statistic*	0.047 (df = 1; 55)
Age: 19–65 years	0.981
	(1.428)
Age: 66–80 years	0.499
	(1.439)
Age: 81–90 years	0.294
	(1.414)
Age: 91–100 years	0.923
	(1.286)
Age: > 100 years	−0.246
	(1.348)
*Observations*^*#*^	70
*R*^*2*^	0.267
*Adjusted R*^*2*^	0.008
*F Statistic*	3.710*** (df = 5; 51)
Proponent of IHPC: General practitioner	0.225
	(0.272)
Proponent IHPC: Residential facilities and other settings	0.321
	(0.194)
*Observations*^*#*^	70
*R2*	0.043
*Adjusted R2*	−0.223
*F Statistic*	1.201 (df = 2; 54)
1 Cohabitant family members	0.004
	(0.124)
2 Cohabitant family members	−0.017
	(0.297)
≥3 Cohabitant family members	−0.090
	(0.543)
*Observations*^*#*^	70
*R2*	0.001
*Adjusted R2*	0.026(df = 3; 53)
*F Statistic*	0.026(df = 3; 53)
Symptoms of the nervous system	−1.517
	(2.374)
Abdominal pain	−1.672
	(2.349)
Chest pain	−0.557
	(2.807)
Dispnea	−1.619
	(2.3489)
Shock	−1.645
	(2.409)
Non-traumatic bleeding	−2.017
	(2.326)
Trauma	−1.527
	(2.357)
Temperature	−1.436
	(2.415)
Rhythm alteration	−1.267
	(2.303)
Other symptoms	−1.702
	(2.317)
Urological symptoms	−1.442
	(2.327)
*Observations*^*#*^	69
*R2*	0.191
*Adjusted R2*	−0.251
*F Statistic*	0.943 (df = 11; 44)

**Notes:** Robust standard errors are given in parentheses under the coefficients,

#The data pool has been modeled as a datapanel, composed by 5 years of average observations over 14 pathologies, which results in 70 overall observations

*** *p* < 0.05

## Discussion

Inferential statistical analysis provided information on the scale of decline in ED visits by showing the annual change in ED visit rates. ED visit rates showed a slight decline in all pathology categories, and this decrease accelerated over the study period. This result may have been facilitated by the type of healthcare system within which our study took place. The Italian National Healthcare System is tax-payer funded, with both public and private providers, and is managed mainly by each of the 21 regions and the autonomous provinces of the country. Each region then has its own Local Health Agencies, which are responsible for ensuring hospital care, care in residential structures, and home care for their population. IHPC is also provided by the National Healthcare System and is officially recognized by the essential levels of care (“livelli essenziali di assistenza”) decree as a benefit guaranteed to all citizens through public resources. The suggestion that our results may be due to a study setting within the Italian healthcare system is in agreement with a systematic review [8] that reported that IHPC events were associated with a significant reduction in ED visits in all studies conducted in countries that have developed strategies for increasing access and quality of care [18, 19].

Although ED visit rates fell for all investigated pathology categories between 2013 and 2017, the scale of change varied across categories. Neoplasms deserve particular attention for two reasons. First, patients with neoplasms received more IHPC events than other patients in our study. This suggests that, although efforts like the introduction of an Italian law in 2010 guaranteeing the right to receive specialist palliative care to citizens with advanced and complex health conditions, especially non-cancer patients, a cancer diagnosis remains one of the most common reasons that patients gain access to IHPC in Italy. This result is supported by the literature: a previous review demonstrated that patients with different characteristics had unequal access to community palliative care services, with cancer patients making the highest use of such care. However, the review did not state whether the variability in the use of community palliative care services was attributable to clinical needs, difficult prognosis, or others factors [20]. Second, ED visit rates declined more slowly among patients with neoplasms than in those with other pathologies. This result is consistent with a previous study that reported that the number of patients in need of ED visits reduced by 59% for patients with chronic medical illness and by 46% for cancer patients [21]. The increased severity of illness, along with tthe fragility of patients, might explain the slow decline of ED visits among cancer patients, but this result may also be partially explained by the need to improve the effectiveness of home care, so that patients enrolled in IHPC can avoid ED visits. Although the Italian healthcare system has extensive and long-standing experience in IHPC for cancer patients, which started in the mid-2000s, the number of Palliative Care Units in Italy has continued to grow. According to the Ministry of Health, their effectiveness still needs to be improved, and the main problem lies with the providers and managers of home care, as they do not guarantee the presence of teams capable of offering 24-h assistance and lack the ability to monitor quality of care [22].

To our knowledge, this is the first study to specifically examine the impact of IHPC on ED visits in patients with different pathologies, including cancer. Previous studies examined whether palliative home care is more effective than usual care at reducing ED visits among patients with cancer, and found that patients receiving palliative home care had fewer ED visits than those without palliative home care (68% versus 79%, *p* = 0.004) [8]; however, no one has evaluated whether improvements in home care outcomes manifest themselves as a reduction in ED visit rates.

According to the results, socio-demographic, health supply, and clinical factors, like age, sex, proponent of IHPC, distance to the nearest ED, and reason for ED visit, are not related to ED visit rates. Not even the presence of a non-family caregiver or an increasing number of cohabitant family members had a significant association with ED visits. The presence of a non-family caregiver was investigated as this presence may reduce the risk of ED visits. Indeed, unfamiliar caregivers are often educated regarding risk, as well as in the monitoring and supervision of patients. Moreover, unfamiliar caregivers are devoted to the care and assistance of patients; their continuous monitoring makes it easier to identify and anticipate the potential risks of adverse outcomes; and they can contact general practitioners to prevent possible acute exacerbations of chronic conditions and adjust standard treatments to individual patients’ needs [23]. The number of cohabitant family members was investigated because southern European countries, such as Italy, are commonly referred to as ‘strong-family-ties countries’ [24]. The strength of family ties is usually highest in families that live together and is discussed in terms of cultural patterns of family loyalties, allegiances, and authority, but it also concerns patterns of intra-generational cohabitation and patterns of support for the elderly and the young. Family relationships can lead to stronger intergenerational solidarity and allow more able members to help more vulnerable members, thereby promoting health enhancement and well-being [25]. The larger a family is, the more people are available to monitor and detect changes in the health status of an ill member and initiate immediate treatment, which may be beneficial in reducing ED use.

The findings of the present study have multiple policy implications. First, home care managers should support the decline in ED visits and use ED only when unavoidable. Teams should work with a holistic perspective around the clock, which should include the best palliation of both physical and psychological symptoms. Palliative care teams should also be sure to offer support through the greater involvement of patients and family members, who must be properly informed and trained on when to use the ED, by providing family members with the skill set necessary to manage an illness and to navigate the healthcare system, and by offering emotional and advocacy support as needed. Previous studies have underlined the complexity of home care, reporting that the resolution of frequent family imbalance and distress must be incorporated into home healthcare services [26, 27]. Second, although the World Health Organization has stated that patients with any advanced progressive disease may benefit from IHPC, our study confirms previous research showing that palliative care services are most often used by cancer patients and are used very little by other patients, despite much evidence showing a need among these patients [28, 29]. Policy makers should take initiatives to improve access to palliative care for all patients with severe disabilities through the strengthening of palliative care networks and the training of teams to guarantee adequate skills [30].

The results and implications of this study must be considered in light of the study’s limitations. The main limitations are those of the databases used and are common to all administrative database studies. Firstly, there are problems related to the quality of the data on IHPC, especially with regard to the possible lack of accuracy and different coding criteria across individuals and institutions. Secondly, the socio-demographic factors have some limitations, as they are often not recorded or require caution regarding their reliability. Even taking these weaknesses into account, these databases are the best available sources, suitable for wide epidemiological studies on the prevalence and incidence of major diagnoses or diseases and for monitoring population trends in the utilization of services.

## Conclusions

Out results show that use of ED continues during IHPC, but the trend showed a reduction over time. As no significant predictive factors were identified, no specific interventions can be recommended to reduce avoidable ED visits. More studies involving other countries are required to support our results, to investigate other prognostic factors related to a continuous need for emergency care, and to increase knowledge in this field.

## Data Availability

The datasets generated and/or analyzed during the current study are not publicly available due data are not public but are available from the corresponding author on reasonable request.

## Abbreviations

IHPC: Integrated palliative home care
ED: Emergency Department
